# Tailoring Photocatalytic Performance of BaTi_5_O_11_ Nanocrystals via Optimizing Sol–Gel Parameters for Efficient Levofloxacin Degradation

**DOI:** 10.3390/gels12080670

**Published:** 2026-07-25

**Authors:** Honghua Wang, Zherui Xing, Xingran Wang, Zhixiong Huang, Dongyun Guo

**Affiliations:** School of Materials Science and Engineering, Wuhan University of Technology, Wuhan 430070, China

**Keywords:** BaTi_5_O_11_ nanocrystals, sol–gel method, processing parameters, photocatalytic performance, levofloxacin photodegradation

## Abstract

The effect of drying, thermal decomposition, and sintering conditions during the sol–gel synthesis of BaTi_5_O_11_ nanocrystals was investigated to optimize levofloxacin (LEV) photodegradation. Sintering emerges as the dominant factor, and BaTi_5_O_11_ nanocrystals synthesized at 700 °C for 120 min exhibit the smallest grain size, highest specific surface area and abundant active sites, achieving 93.2% LEV degradation within 30 min under UV irradiation. In contrast, excessive sintering temperatures or time induce grain coarsening and size homogenization, which reduce surface area and active sites, thereby impairing photocatalytic performance. The optimized nanocrystals also efficiently degrade other antibiotic pollutants, including ciprofloxacin, norfloxacin, and tetracycline. Radical trapping experiments confirm that •OH is the primary reactive species. Photoluminescence and photoelectrochemical analyses reveal a competition between grain size variation and charge carrier dynamics; however, photocatalytic degradation underscores the dominant role of surface-active sites and specific surface area. Kelvin probe force microscopy (KPFM) further corroborates efficient charge separation, showing a cross-line contact potential difference (ΔV_CPD_) of approximately 90 mV, indicative of facile hole migration to the crystal surface. Collectively, these findings elucidate the processing–microstructure–property relationships in BaTi_5_O_11_ nanocrystals and provide a robust basis for the rational design of high-performance photocatalytic systems for antibiotic pollutant remediation.

## 1. Introduction

Antibiotics serve as a crucial tool for combating bacterial infections and have thus been widely employed in medical practice. Their extensive use, however, has led to the pervasive release of antibiotic residues into aquatic environments, including municipal wastewater, groundwater, and surface water [1], contributing to microecological disruption, the proliferation of drug-resistant pathogens, and potential risks to human health [2]. Levofloxacin (LEV), a broad-spectrum fluoroquinolone antibiotic, is commonly prescribed for severe bacterial infections [3]. Owing to its limited metabolic conversion, LEV resists direct biodegradation and persists in natural water systems. Since Müller’s 1969 demonstration of the photocatalytic degradation of isopropanol over ZnO under ultraviolet (UV) light [4], a diverse array of advanced photocatalytic materials—such as S-scheme heterojunctions, metal–organic frameworks (MOFs), and quantum dot-modified perovskites—has been developed for applications including hydrogen evolution [5,6,7], CO_2_ photoreduction [8,9], organic pollutant degradation [10] and H_2_O_2_ photosynthesis [11]. In response to the challenge of antibiotic contamination, advanced oxidation processes (AOPs) have been increasingly adopted for the efficient removal of LEV [12]. Photocatalysis has been considered one of the most promising methods for degrading LEV due to its minimal or no chemical additives, simple process, high efficiency and environmental compatibility [13,14]. Within the spectrum of photocatalyst materials, single-crystal semiconductors have attracted considerable interest owing to their well-defined morphology, low defect density, and capacity to expose specific crystal facets [15]. While wide-bandgap single-crystal semiconductors generate photogenerated charge carriers with strong redox potentials under UV excitation [16,17], these carriers are subject to rapid recombination driven by strong Coulombic interactions [18]. Introducing an internal electric field within the crystal lattice has proven effective in promoting charge separation [19]. BaTi_5_O_11_, a dielectric material characterized by a wide bandgap, contains distorted TiO_6_ octahedra that induce spontaneous polarization. This polarization establishes built-in electric fields that facilitate efficient charge migration over short distances [20], positioning BaTi_5_O_11_ nanocrystals as compelling candidates for photocatalytic applications.

In our previous work, BaTi_5_O_11_ nanocrystals synthesized via the sol–gel method demonstrated excellent photocatalytic activity toward methylene blue (MB), achieving a photocatalytic degradation of 99% within 20 min and a reaction rate constant of 0.28 min^−1^ [21]. This success suggests that BaTi_5_O_11_ may also be effective for the photocatalytic degradation of LEV. As a well-established wet-chemical technique, the sol–gel method offers numerous advantages, including the scalable production of homogeneous materials with high purity, large specific surface area, high porosity, and enhanced reactivity. It is cost-effective, operationally straightforward, and enables fine control over particle size and morphology [22,23,24]. Previous studies have identified sintering temperature and solvent type as critical parameters affecting crystallite size, surface area, and photocatalytic performance of BaTi_5_O_11_ nanocrystals [21,25,26]. Other investigations have further demonstrated that precursor concentration, pH value, and reaction temperature exert substantial influence over grain dimensions, morphology, oxygen vacancy concentration, and lattice defects [27,28]. The complete sol–gel synthesis route encompasses hydrolysis, polycondensation, solvent evaporation, pyrolysis of cross-linked metal alkoxides, and sintering for crystallization [22]. Parameters such as drying temperature, as well as pyrolysis temperature and duration, have also been shown to critically affect the morphology and functional properties of the resultant materials [28,29,30].

The rational design of synthetic pathways is therefore essential for obtaining BaTi_5_O_11_ nanomaterials with tailored microstructural features, particularly grain size and Brunauer–Emmett–Teller (BET) surface area. During the solvent evaporation stage, removal of the solvent yields a xerogel with a three-dimensional network structure. Excessively low evaporation temperatures may leave residual solvent within the xerogel, leading to carbonaceous deposits on the product surface; conversely, overly high evaporation temperatures promote excessive cross-linking among metal alkoxide species [31], resulting in larger particle sizes, diminished surface area, and reduced reactivity [32]. Thus, precise control over evaporation temperature is instrumental in optimizing the photocatalytic properties of the final crystals. In the subsequent pyrolysis step, the cross-linked organic metal alkoxides decompose to form amorphous Ba–Ti–O powders. Both the pyrolysis temperature and duration critically influence residual organic content and powder morphology. During sintering, elevated temperatures markedly affect crystalline morphology, grain size and distribution [26], crystallinity [33], bandgap energy [34] and other physicochemical characteristics [35]. Furthermore, higher sintering temperatures intensify crystal agglomeration, which may compromise photocatalytic performance.

In the present study, BaTi_5_O_11_ nanocrystals were synthesized by the sol–gel method. The effects of drying temperature (250, 260 or 270 °C), pyrolysis temperature (300, 350, 400 or 450 °C) and time (60, 120, 180 or 240 min), and sintering temperature (650, 700, 750, 800, or 850 °C) and time (60, 120, 180, or 240 min) were systematically investigated to optimize microstructural properties and enhance the photocatalytic degradation of LEV, with the goal of achieving superior antibiotic removal. Moreover, the underlying photocatalytic mechanism was comprehensively elucidated through analysis of band-edge positions, reactive radical species, photoelectrochemical behavior, and interfacial charge transfer dynamics. This integrated approach provides a thorough understanding of the factors governing the photocatalytic performance of BaTi_5_O_11_.

## 2. Results and Discussions

### 2.1. Drying Temperature

In this experiment, the Ba–Ti precursors were dried at different temperatures (250, 260 and 270 °C) for 180 min, then thermally decomposed at 400 °C for 180 min, and finally sintered at 750 °C for 180 min. The obtained products were characterized by XRD and SEM, and their photocatalytic performance was evaluated.

**Figure 1:**
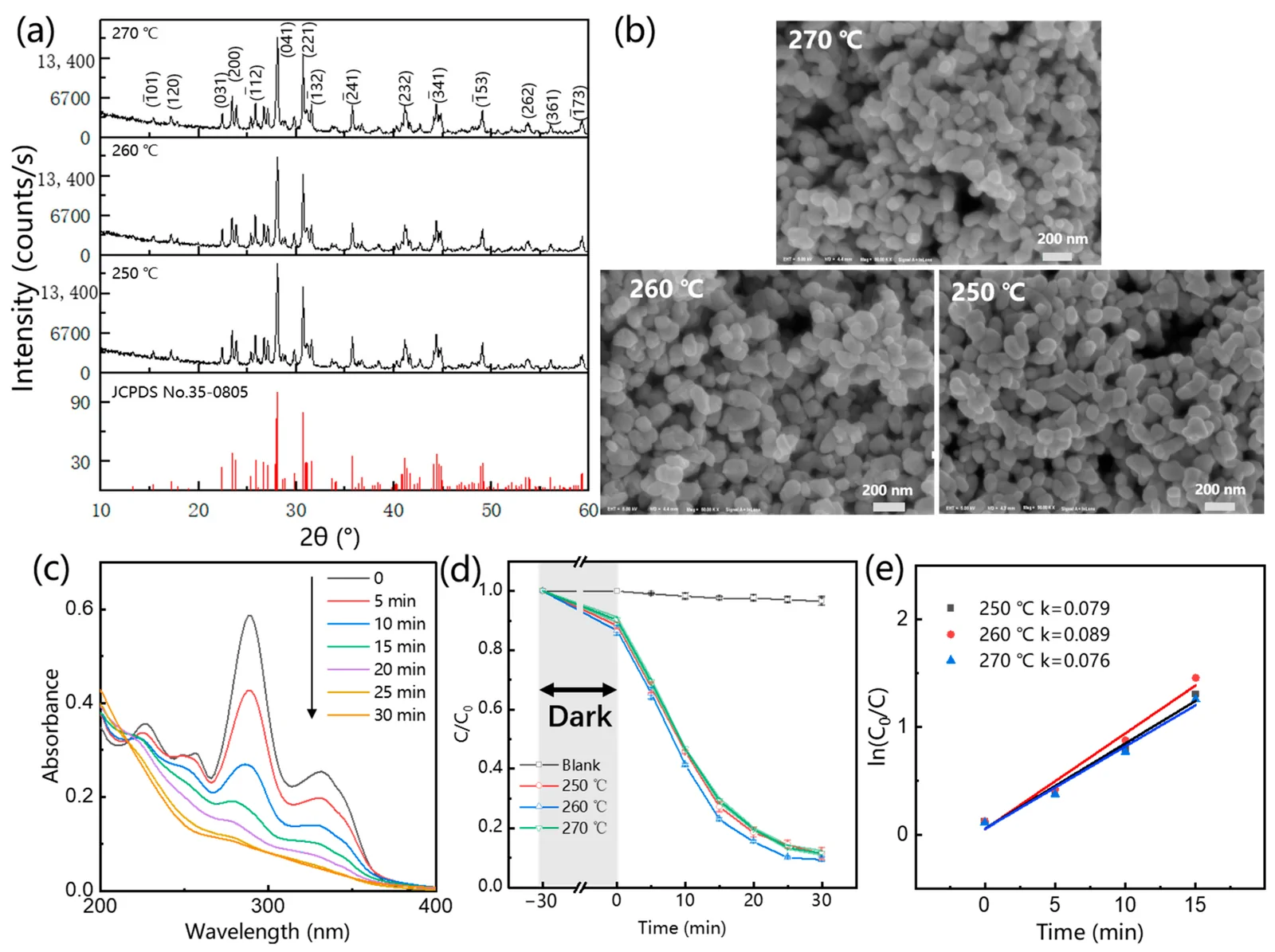
BaTi_5_O_11_ samples synthesized at different drying temperatures: (**a**) their XRD patterns, (**b**) SEM images, Imaging parameters: EHT = 5.00 kV; WD = 4.3 mm; Mag = 50.00 KX; Signal A = InLens (250 °C). EHT = 5.00 kV; WD = 4.4 mm; Mag = 50.00 KX; Signal A = InLens (260 °C and 270 °C). (**c**) UV–vis absorption spectra of LEV solutions photodegraded at different times by BaTi_5_O_11_ nanocrystals dried at 260 °C, (**d**) time-dependent photocatalytic degradations, and (**e**) their pseudo-first-order kinetics for LEV photodegradation by BaTi_5_O_11_ nanocrystals.

Figure 1a shows the XRD results of these products. All XRD patterns reveal sharp diffraction peaks corresponding to a pure monoclinic BaTi_5_O_11_ phase (JCPDS No. 35-0805), with no detectable impurity. The patterns are fully indexed to the standard reference, confirming the phase purity and crystallinity. Importantly, the diffraction profiles of samples prepared at different drying temperatures are nearly identical, indicating that the monoclinic phase formation is unaffected by the tested temperature range (250–270 °C). Consistent with the XRD data, the SEM images (Figure 1b) demonstrate uniform grain morphologies across all samples, with an average grain size of about 100 nm. These observations collectively suggest that variations in drying temperature within the studied range have no observable effect on the phase composition or microstructure of the BaTi_5_O_11_ nanocrystals.

The photocatalytic activity of BaTi_5_O_11_ nanocrystals was evaluated by monitoring the UV light-driven degradation of LEV. The concentration of LEV was quantified by measuring the UV–vis absorption intensity at its characteristic wavelength of 288 nm, where a linear correlation with concentration was observed. Quantification of LEV concentration was performed via UV–vis absorption measurements at its characteristic wavelength (288 nm), which exhibited a linear calibration curve. Figure 1c shows the time-dependent UV–vis spectra (200–400 nm) of LEV during degradation catalyzed by BaTi_5_O_11_ nanocrystals dried at 260 °C. A continuous decline in the 288 nm absorption peak intensity confirms the effective degradation of LEV. To evaluate the temperature dependence, Figure 1d compares the normalized LEV concentration (C/C_0_, where C_0_ and C denote the initial and time-dependent concentrations, respectively) for BaTi_5_O_11_ nanocrystals dried at 250, 260, and 270 °C. Prior to irradiation, the BaTi_5_O_11_ photocatalyst–LEV mixture was stirred in the dark for 30 min to achieve adsorption–desorption equilibrium. Control experiments revealed negligible LEV self-degradation (4.8% after 30 min) under UV-light irradiation without the catalyst, highlighting the essential role of BaTi_5_O_11_ nanocrystals. Strikingly, the BaTi_5_O_11_ nanocrystals achieved high photocatalytic degradations of 88.4%, 90.0%, and 89.1% at 250, 260, and 270 °C, respectively, within 30 min of UV-light irradiation. The minimal variation in degradation (<2%) indicates that drying temperature has limited impact on photocatalytic performance. This consistency suggests that temperatures near the solvent boiling point (260 °C) are sufficient to produce active nanocrystals. The marginally enhanced activity at 260 °C may arise from its proximity to the solvent boiling point, promoting complete solvent removal and thereby optimizing crystallinity [36]. Therefore, in the next study, the drying parameters were set to 260 °C for 180 min.

### 2.2. Thermal Decomposition Process

In this study, the Ba–Ti precursors were dried at 260 °C for 180 min, followed by thermal decomposition at varying temperatures (300, 350, 400, and 450 °C) for various times (60, 120, 180 and 240 min), and finally sintered at 750 °C for 180 min. The obtained products were characterized by XRD and SEM, and their photocatalytic performance was evaluated.

**Figure 2:**
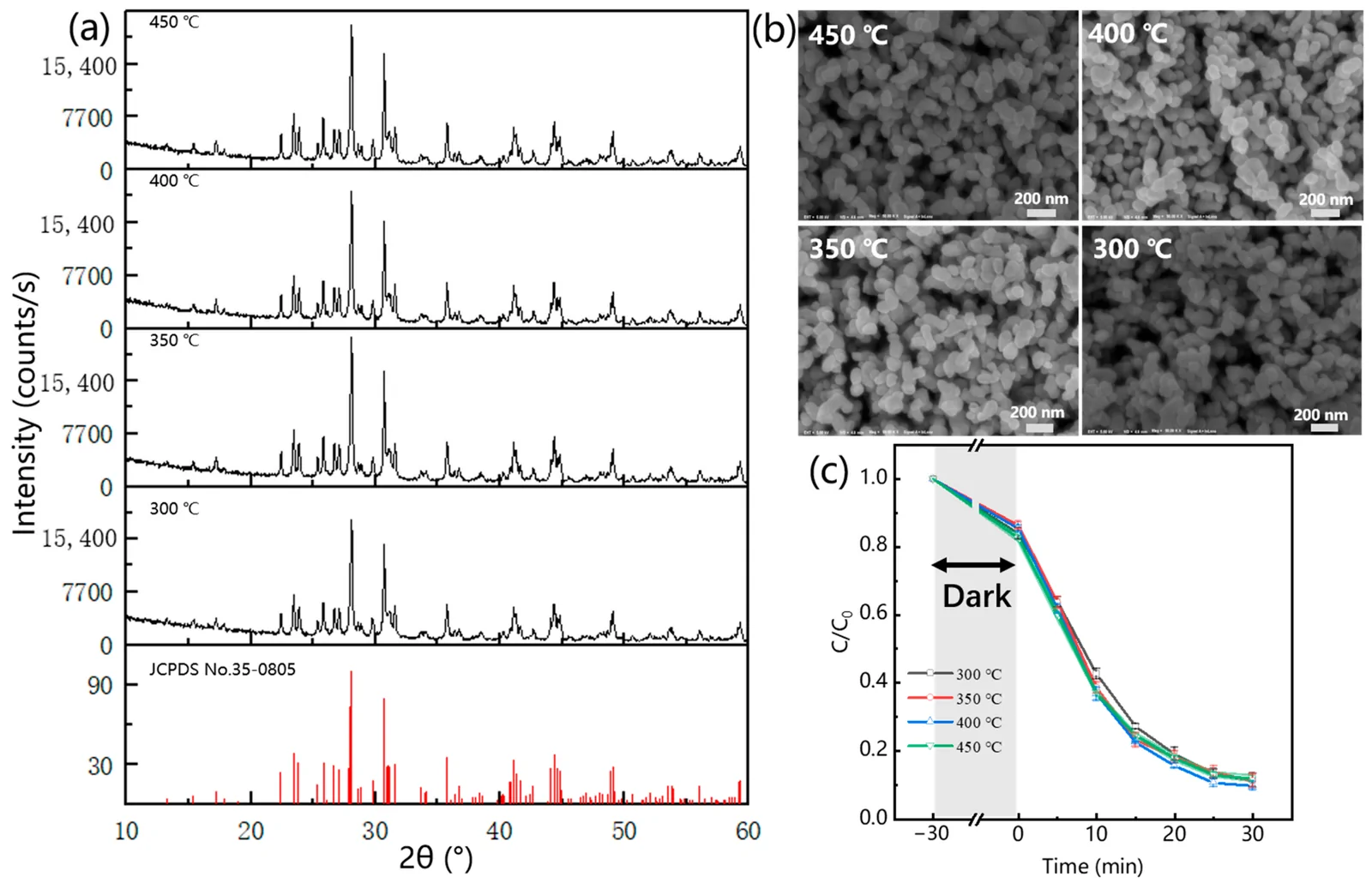
BaTi_5_O_11_ samples synthesized at different thermal decomposition temperatures for 180 min: (**a**) XRD patterns, (**b**) morphologies, all imaging parameters were identical: EHT = 5.00 kV; WD = 4.6 mm; Mag = 50.00 KX; Signal A = InLens (**c**) time-dependent photocatalytic degradations for LEV in the presence of BaTi_5_O_11_ nanocrystals.

Figure 2a shows the XRD patterns of BaTi_5_O_11_ samples synthesized under different thermal decomposition temperatures for 180 min. All XRD patterns exhibit a pure monoclinic BaTi_5_O_11_ phase without detectable impurity. Notably, no significant differences are observed in the XRD patterns of samples prepared at varying thermal decomposition temperatures, suggesting that the selected temperature range does not alter the crystalline phase formation. As shown in Figure 2b, consistent with the XRD results (Figure 2a), the SEM images reveal uniform particle morphologies with an average grain size of about 100 nm, further supporting that the thermal decomposition temperature has negligible influence on either the crystal structure or the morphology. These findings collectively indicate that the monoclinic phase and nanoscale morphology of BaTi_5_O_11_ are robust against thermal decomposition temperature variations under the tested conditions.

Figure 2c shows the normalized concentration of LEV as a function of UV irradiation time for BaTi_5_O_11_ nanocrystals synthesized at thermal decomposition temperatures ranging from 300 to 450 °C. The incorporation of BaTi_5_O_11_ nanocrystals significantly enhanced the photocatalytic degradation of LEV, with degradation rates reaching 86.4%, 88.2%, 89.0%, and 87.2% after 30 min of UV exposure for samples prepared at 300, 350, 400, and 450 °C, respectively. Although the differences in degradation performance among the samples were marginal, the BaTi_5_O_11_ nanocrystals synthesized at 350 and 400 °C exhibited slightly superior photocatalytic activity compared to those produced at 300 and 450 °C. This temperature-dependent behavior can be linked to structural evolution during thermal decomposition process. At lower temperature (300 °C), incomplete decomposition of cross-linked organic–metal alkoxides likely leaves residual organic species, which may block active sites and impede photocatalytic reactions [37]. Conversely, at elevated temperature (450 °C), rapid solvent removal from the gel induces excessive network shrinkage, reducing porosity and promoting uncontrolled grain growth. This process diminishes the surface area and availability of reactive sites, ultimately lowering photocatalytic degradation. In contrast, intermediate temperatures (350–400 °C) balance structural integrity and active site accessibility, optimizing photocatalytic performance. Therefore, for the following experiment, the thermal decomposition temperature was set to 400 °C.

**Figure 3:**
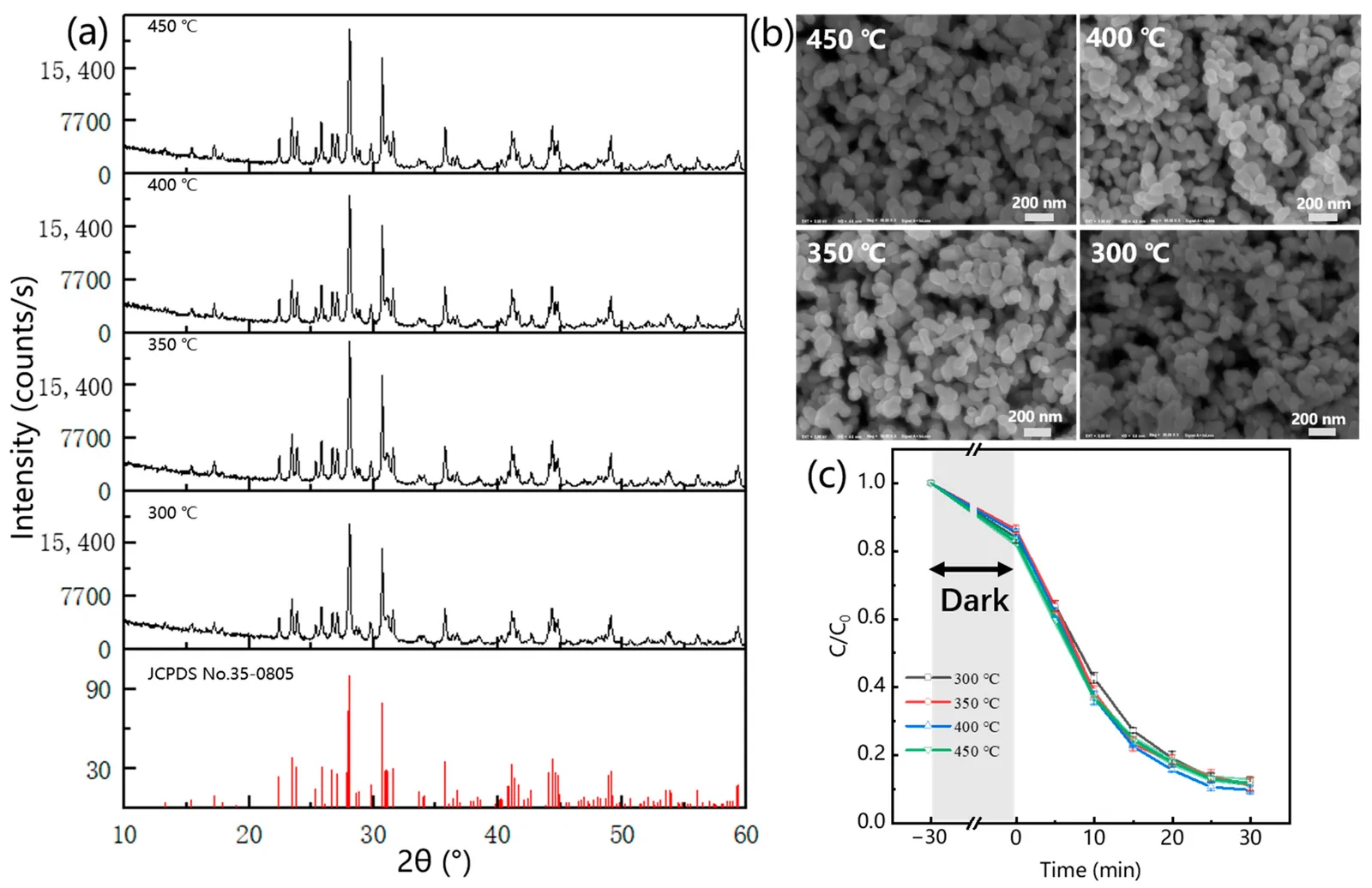
BaTi_5_O_11_ samples synthesized at 400 °C with different thermal decomposition times: (**a**) XRD patterns, (**b**) morphologies, imaging parameters: EHT = 5.00 KV; WD = 4.4 mm; Mag = 50.00 KX; Signal A = InLens (60, 120, 180 min). EHT = 5.00 KV; WD = 4.5 mm; Mag = 50.00 KX; Signal A = InLens (240 min). (**c**) time-dependent photocatalytic degradations for LEV in the presence of BaTi_5_O_11_ nanocrystals.

Figure 3a presents the XRD patterns of BaTi_5_O_11_ samples synthesized at 400 °C with varying thermal decomposition times. All XRD patterns correspond to a pure monoclinic BaTi_5_O_11_ phase, with no detectable impurity, and are well indexed to the standard pattern of monoclinic BaTi_5_O_11_. Notably, the diffraction peaks of all samples align closely with the monoclinic BaTi_5_O_11_ phase. Furthermore, the XRD patterns obtained under different thermal decomposition times show striking similarity, implying that the tested time range does not disrupt the phase formation. Figure 3b demonstrates the SEM images. The uniform particle morphologies are observed with an average grain size of about 100 nm, regardless of the thermal decomposition time, which is consistent with the XRD results (Figure 3a). These observations collectively suggest that variations in thermal decomposition time have negligible influence on the phase purity, crystal structure, and morphology of the BaTi_5_O_11_ samples under the applied synthesis conditions.

Figure 3c shows the normalized LEV concentration as a function of UV irradiation time for BaTi_5_O_11_ nanocrystals synthesized at 400 °C with different thermal decomposition time. After 30 min of UV-light irradiation, the photocatalytic degradations of BaTi_5_O_11_ nanocrystal catalysts prepared at 400 °C for different thermal decomposition times were 85.6%, 89.0%, 88.2%, and 87.9%, respectively. The photocatalytic degradation of the BaTi_5_O_11_ nanocrystal catalyst prepared with a thermal decomposition time of 60 min was significantly lower than those prepared with other thermal decomposition time. This was mainly due to the insufficient removal of organic substances caused by the shorter thermal decomposition time. The residual organic matter may have affected the photocatalytic performance of the BaTi_5_O_11_ nanocrystals. The BaTi_5_O_11_ nanocrystal catalysts prepared with thermal decomposition time of 120 min or longer all exhibited good photocatalytic performance. Considering all factors, a thermal decomposition time of 120 min was selected to conduct further research.

### 2.3. Sintering Process

In the sintering process, the Ba–Ti precursors were dried at 260 °C for 180 min, followed by thermal decomposition at 400 °C for 120 min, and finally sintered at different temperatures (600, 700, 750, 800 and 850 °C) for a certain amount of time (60, 120, 180 and 240 min). The obtained products were characterized by XRD and SEM, and their photocatalytic performance was evaluated.

**Figure 4:**
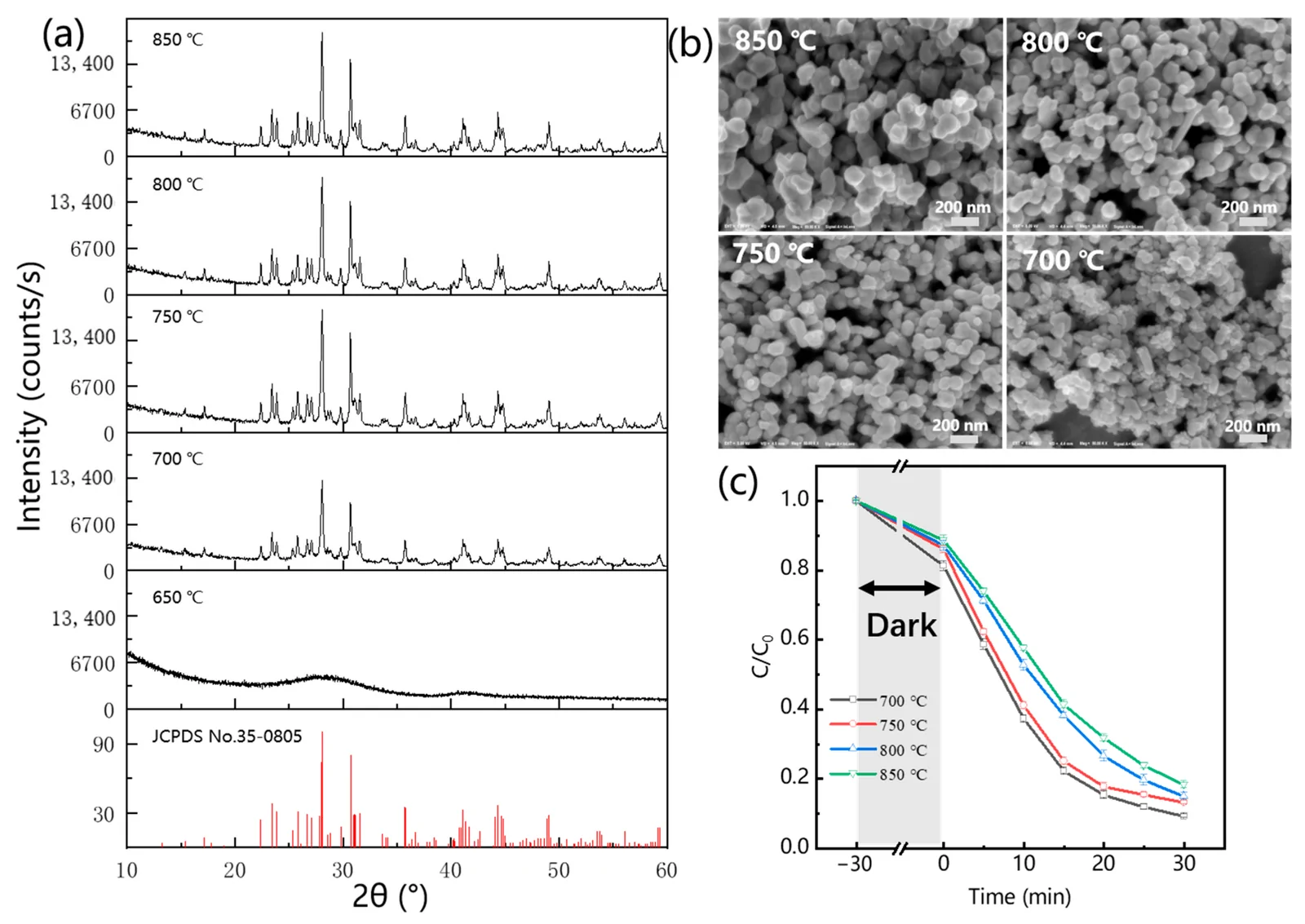
BaTi_5_O_11_ samples sintered at different temperatures: (**a**) XRD patterns, (**b**) morphologies, imaging parameters: EHT = 5.00 KV; WD = 4.4 mm; Mag = 50.00 KX; Signal A = InLens (700 °C and 800 °C). EHT = 5.00 KV; WD = 4.5 mm; Mag = 50.00 KX; Signal A = InLens (750 °C and 850 °C). (**c**) time-dependent photocatalytic degradations for LEV by BaTi_5_O_11_ nanocrystals.

Figure 4a shows the XRD results of BaTi_5_O_11_ samples sintered at 650–850 °C for 180 min. The sample sintered at 650 °C exhibited amorphous characteristics, as evidenced by the absence of diffraction peaks. As the sintering temperature increased to 700 °C, sharp diffraction peaks were observed, which were indexed to the standard monoclinic BaTi_5_O_11_ phase, confirming that the pure BaTi_5_O_11_ phase was obtained. With increasing the sintering temperature from 700 °C to 750 °C, an obvious enhancement in peak intensity occurred, while the XRD patterns remained consistent from 750 °C to 850 °C. These observations demonstrate the temperature-dependent crystallization behavior of the monoclinic BaTi_5_O_11_ phase, with 700 °C representing the critical threshold for phase formation. As shown in Figure 4b, the SEM analysis revealed the progressive microstructural evolution with increasing sintering temperature. At 700 °C, nanoscale grains (10–100 nm) with high surface area morphology were observed, favorable for catalytic applications through enhanced active site availability. Subsequent temperature elevation promoted grain growth and uniformity [25]. This microstructural coarsening correlates with thermally activated atomic rearrangement processes that reduce lattice defects and improve crystallinity. The observed 50 °C reduction in crystallization temperature compared to our previous study using acetylacetone solvent (750 °C) [26] highlights the critical role of precursor coordination chemistry. Acetylacetone combines with Ba^2+^ and Ti^4+^ ions through chemical bonding to form stable complexes, while PEG-200 acts as a dispersant, forming weak physical interactions with Ba^2+^ and Ti^4+^ ions. Moreover, as a macromolecular compound, PEG-200 undergoes thermal decomposition and volatilizes at high temperatures. When this decomposition process occurs, it often leaves behind pores, which further facilitates the formation of porous BaTi_5_O_11_ nanocrystals. This mechanistic difference underscores the importance of solvent selection in controlling nucleation kinetics and final microstructure. This strong coordination significantly reduces the reactivity of the precursor, requiring a higher temperature to break the coordination bond, thereby increasing the crystallization temperature. On the contrary, weak coordination solvents such as alcohols have a relatively weak coordination effect on the precursors, which is conducive to the progress of hydrolytic and condensation reactions and enables crystallization at lower temperatures.

Figure 4c illustrates the normalized LEV concentration versus irradiation time for BaTi_5_O_11_ nanocrystals. During the pre-irradiation adsorption phase (30 min dark stirring), the BaTi_5_O_11_ nanocrystal photocatalyst sintered at 700 °C exhibited the highest LEV adsorption capacity (18%). The addition of BaTi_5_O_11_ nanocrystal photocatalysts significantly enhanced LEV degradation, with efficiencies reaching 90.2%, 87.3%, 85.0%, and 82.7% after 30 min irradiation for BaTi_5_O_11_ samples sintered at 700, 750, 800, and 850 °C, respectively. The superior photocatalytic performance and adsorption capability of the BaTi_5_O_11_ sample sintered at 700 °C were attributed to its increased density of active surface sites and larger specific surface area, respectively.

**Figure 5:**
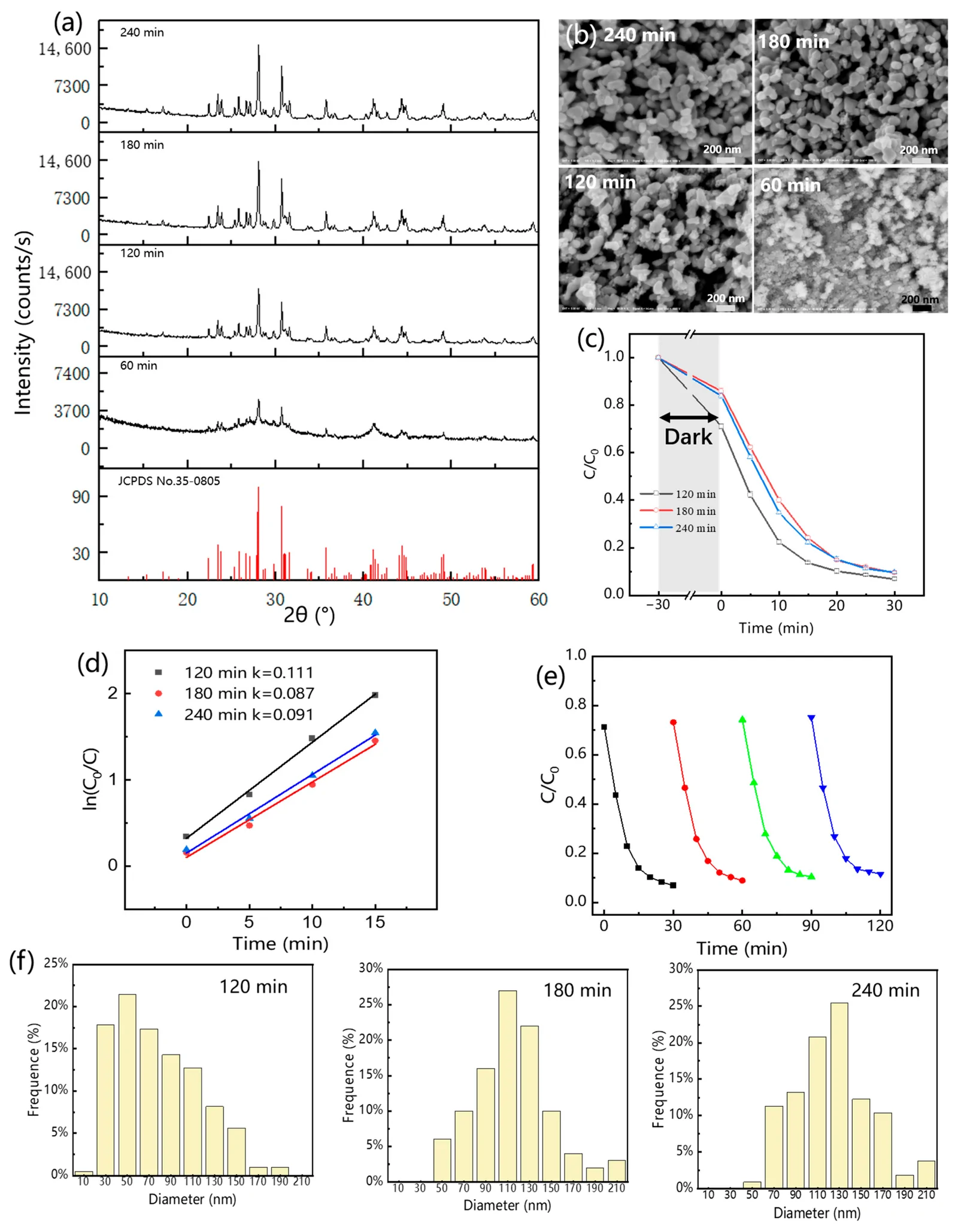
BaTi_5_O_11_ samples synthesized at 700 °C for different lengths of time: (**a**) XRD patterns, (**b**) morphologies, imaging parameters: EHT = 5.00 KV; WD = 5.1 mm; Mag = 50.00 KX; Signal A = InLens (60 min and 180 min). EHT = 5.00 KV; WD = 5.0 mm; Mag = 50.00 KX; Signal A = InLens (120 min). EHT = 5.00 KV; WD = 5.2 mm; Mag = 50.00 KX; Signal A = InLens (240 min). (**c**) time-dependent photocatalytic degradations for LEV in the presence of BaTi_5_O_11_ nanocrystals, (**d**) their pseudo-first-order kinetics for LEV photodegradation, (**e**) photocatalytic cycling performance of BaTi_5_O_11_ nanocrystals with sintering time of 120 min for LEV degradation, and (**f**) grain size distribution.

Figure 5a displays the XRD results of the BaTi_5_O_11_ samples sintered at 700 °C for different holding times (60, 120, 180, and 240 min). When the sintering time was 60 min, only several weak peaks were observed due to the insufficient crystallization. With increasing the sintering time to 120 min, the peak intensity obviously increased, and all XRD peaks were well indexed to the standard monoclinic BaTi_5_O_11_ phase, indicating the formation of monoclinic BaTi_5_O_11_ phase. As the sintering time increased from 120 to 240 min, the peak intensity also increased due to the enhanced crystallization. As shown in Figure 5b, irregular grains were observed due to insufficient crystallization when the sintering time was only 60 min. With increasing sintering time, the grain size became uniform, and the grain sizes ranged from 50 nm to 100 nm, indicating a higher specific surface area and providing more active sites to enhance degradation performance [38].

Figure 5c plots the normalized LEV concentration versus irradiation time for BaTi_5_O_11_ nanocrystals. The addition of BaTi_5_O_11_ nanocrystal photocatalysts led to significant LEV degradation. After 30 min of UV light irradiation, the photocatalytic degradations for BaTi_5_O_11_ nanocrystal photocatalysts synthesized at sintering time of 120, 180, and 240 min were 93.2%,92.1% and 91.9%, respectively. After stirring in darkness for 30 min to establish adsorption–desorption equilibrium, the BaTi_5_O_11_ nanocrystal photocatalyst with a sintering time of 120 min had the highest adsorption of 29% for LEV, which also was ascribed to the higher specific surface area. The BaTi_5_O_11_ nanocrystal photocatalyst sintering for 120 min exhibited the highest *k* value of 0.111 min^−1^, as shown in Figure 5d. The photocatalytic cycling performance of BaTi_5_O_11_ nanocrystals is shown in Figure 5e. It retained a high LEV photocatalytic degradation of 90.1% even after four consecutive cycles, confirming the excellent reusability and stability of the BaTi_5_O_11_ nanocrystal photocatalyst synthesized under the optimized process parameters. Figure 5f shows the grain size distribution of BaTi_5_O_11_ nanocrystal synthesized at 700 °C with different lengths of time. BaTi_5_O_11_ nanocrystal synthesized for 120 min had the smallest grain size, and the grain size increased with extended sintering time. In our previous study [21], the specific surface area increased with decreased grain size, which is consistent with the photocatalytic degradation results.

**Figure 6:**
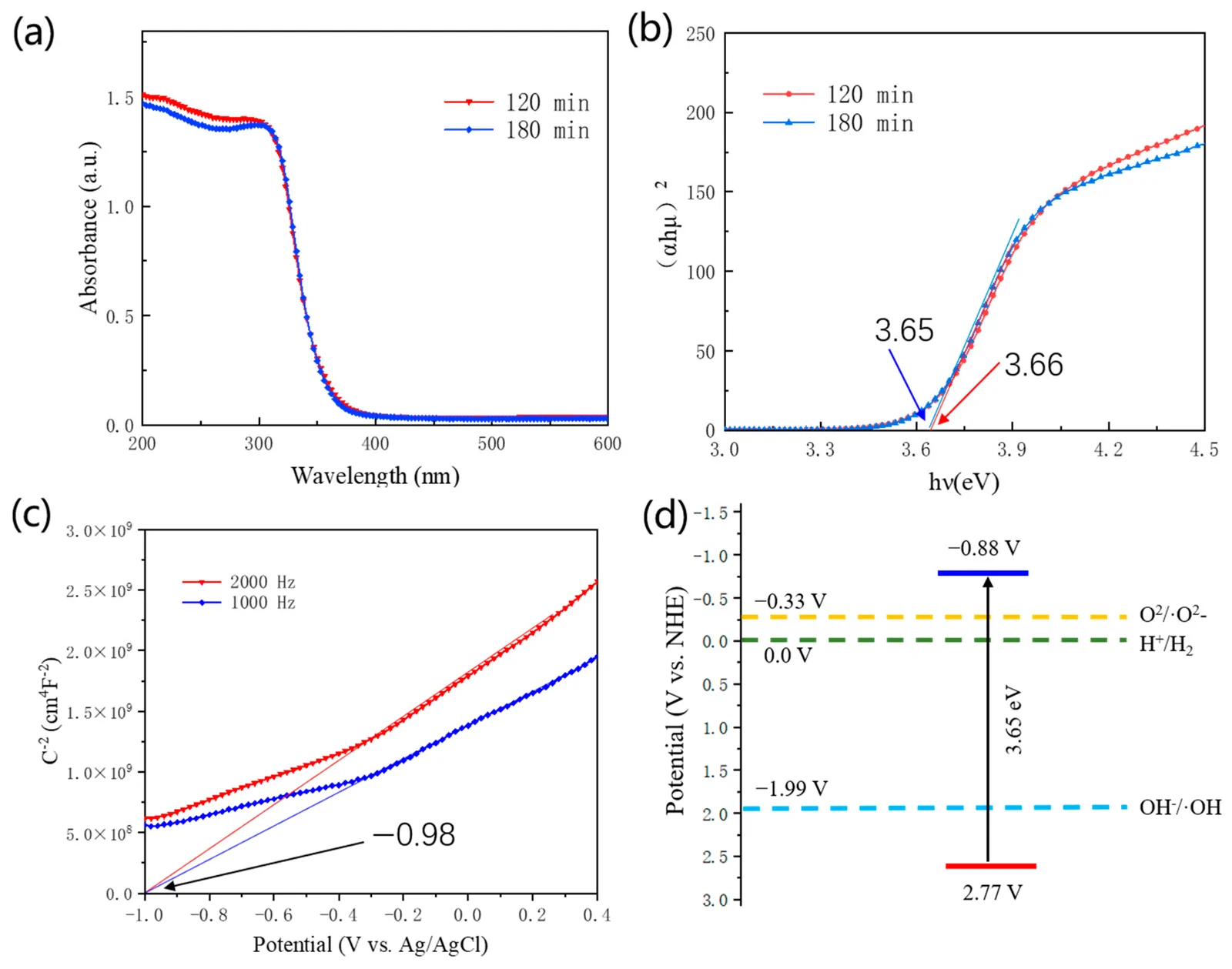
(**a**) UV–vis diffuse reflectance spectra of BaTi_5_O_11_ nanocrystals synthesized at 700 °C for different lengths of time, and (**b**) their transformed Tauc plots, (**c**) Mott–Schottky plots of BaTi_5_O_11_ nanocrystals synthesized at 700 °C for 120 min, and (**d**) their corresponding band structure diagram.

The optical absorption properties of BaTi_5_O_11_ nanocrystals synthesized at 700 °C for 120 min and 180 min were characterized using the UV–vis diffuse reflectance spectroscopy in the wavelength range of 200–600 nm. As shown in Figure 6a, the BaTi_5_O_11_ nanocrystals showed strong UV absorption. The optical band gap (*E*_g_) was calculated from the reflectance data using Equation (1).

(*αhν*)*^n^* = *A* (*hν* − *E_g_*) (1)

where *α* is the absorption coefficient, *hν* is the photon energy and *A* is a constant. As the BaTi_5_O_11_ compound is a direct bandgap semiconductor [20], *n* is equal to two. Figure 6b shows the corresponding Tauc plots of (*αhv*)^2^ vs. *hv*. The calculated *E_g_* values of BaTi_5_O_11_ nanocrystals synthesized at 700 °C for 120 min and 180 min were 3.66 and 3.65 eV, respectively, attributed to the structural ordering and quantum confinement effects. To explore their electronic band structure, Mott–Schottky plot analysis was performed to determine the flat-band potential, which was obtained from extrapolating the slope and intercept with the potential axis. As shown in Figure 6c, the positive slope of the quasi-linear response and negative x-intercept at −0.98 V vs. Ag/AgCl indicate that BaTi_5_O_11_ nanocrystalline is an n-type semiconductor [7]. The value −0.98 V of the negative x-intercept is the flat-band potential. For the n-type semiconductor, the conduction band (CB) is approximately 0.1 V lower than the flat-band potential [39]. As a result, the CB position of BaTi_5_O_11_ nanocrystals was estimated to be −0.88 V vs. the normal hydrogen electrode (NHE), and the valence band (VB) position was 2.77 V according to Equation (2).

*E*_CB_ = *E*_VB_ − *E*_g_ (2)

### 2.4. Photocatalytic Universality and Radical Active Species

**Figure 7:**
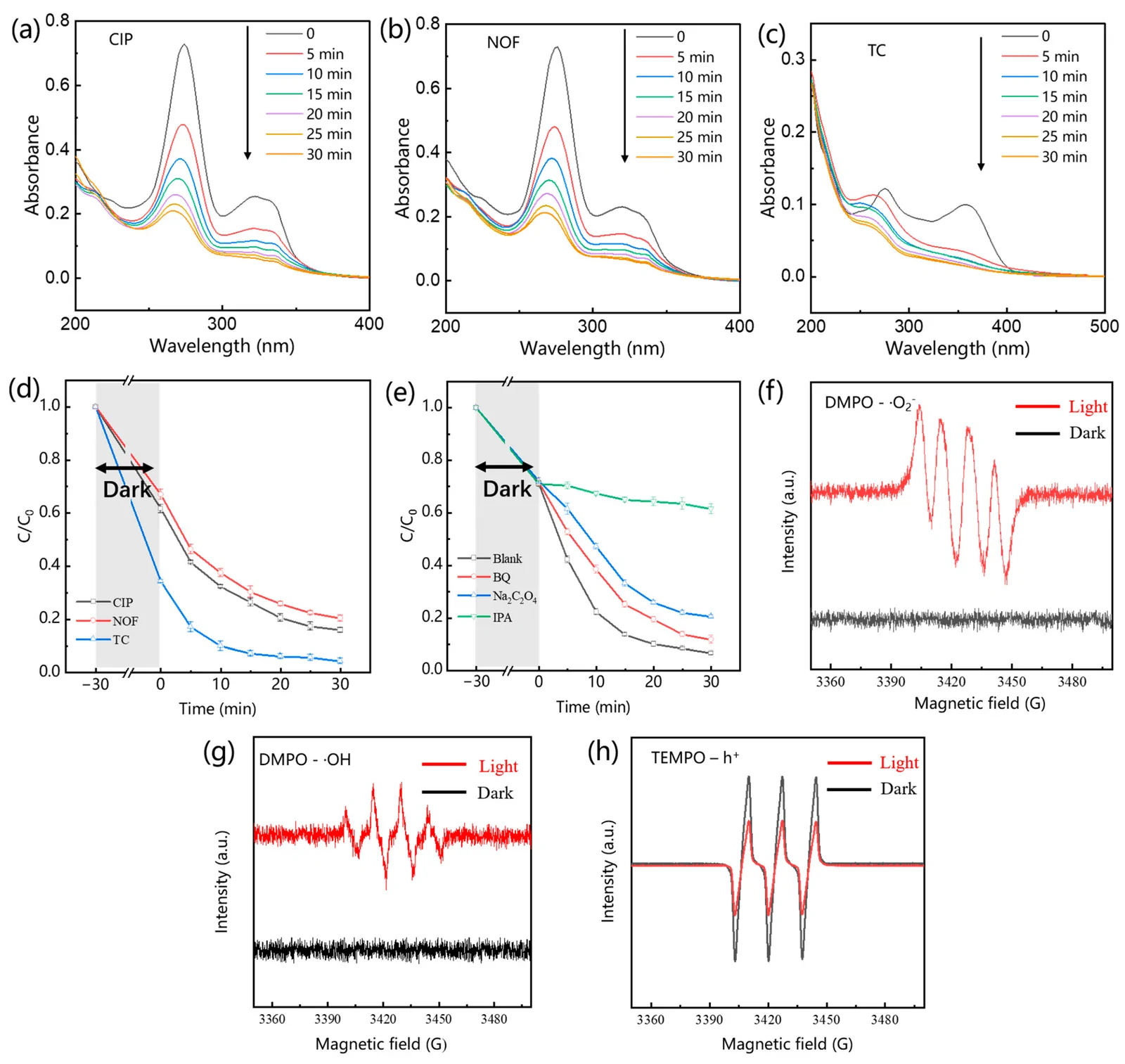
BaTi_5_O_11_ nanocrystals dried at 260 °C for 180 min, thermally decomposed at 400 °C for 120 min, and sintered at 700 °C for 120 min: UV–vis absorption spectra of (**a**) CIP, (**b**) NOF and (**c**) TC solutions photodegraded for different lengths of time by BaTi_5_O_11_ nanocrystals; (**d**) time-dependent photocatalytic degradations for CIP, NOF and TC in the presence of BaTi_5_O_11_ nanocrystals; (**e**) active species trapping experiment of LEV degradation reaction over various scavengers (BQ, IPA and Na_2_C_2_O_4_) in the presence of BaTi_5_O_11_ photocatalyst; and ESR spectra of •O_2_^−^ (**f**), •OH (**g**) and h^+^ (**h**) of BaTi_5_O_11_ under irradiation.

Based on the above research, as they were dried at 260 °C for 180 min, thermally decomposed at 400 °C for 120 min, and sintered at 700 °C for 120 min, the BaTi_5_O_11_ nanocrystals showed the best photocatalytic performance for LEV degradation. Their photocatalytic performance was evaluated through the photodegradation of typical antibiotic pollutants, including ciprofloxacin (CIP), norfloxacin (NOF) and tetracycline (TC) under UV light irradiation. The antibiotic concentrations were determined from the UV–vis absorption intensities at their characteristic wavelengths of 272 nm for CIP, 273 nm for NOF and 357 nm for TC, which exhibited a linear relationship with concentration. Figure 7a–c present the time-dependent UV–vis absorption spectra of antibiotic solutions during degradation catalyzed by the BaTi_5_O_11_ nanocrystals. The characteristic absorption peaks at 272 nm for CIP, 273 nm for NOF and 357 nm for TC all decreased rapidly with increasing irradiation time, indicating efficient antibiotic degradation. Figure 7d shows the *C*/*Co* plots of CIP, NOF and TC solutions in the presence of BaTi_5_O_11_ nanocrystals vs. irradiation time. After 30 min UV-light irradiation, the photocatalytic degradations of CIP, NOF and TC were 83.2%,78.9%, and 95.1%, respectively. The BaTi_5_O_11_ nanocrystals demonstrated high-efficiency photodegradation of antibiotic pollutants, which indicate that the BaTi_5_O_11_ nanocrystals have promising applications in the treatment of wastewater contaminated with antibiotics such as LEV, CIP, NOF and TC.

To explore the photocatalytic degradation mechanism of LEV degradation, radical capture experiments were conducted to identify the free radical species involved under UV irradiation. Benzoquinone (BQ), isopropanol (IPA), and sodium oxalate (Na_2_C_2_O_4_) were employed as scavengers for superoxide radicals (•O_2_^−^), hydroxyl radicals (•OH) and photogenerated holes (h^+^), respectively. Figure 7e presents the time-dependent photocatalytic degradation of LEV in the presence of BaTi_5_O_11_ nanocrystals sintered at 700 °C for 120 min, both with and without the various scavengers. After 30 min UV irradiation, the photocatalytic degradation decreased dramatically with the addition of IPA, indicating that •OH radicals served as the primary active species responsible for LEV degradation. Meanwhile, the photocatalytic degradations decreased to 87.8% and 78.5% upon the addition of BQ and Na_2_C_2_O_4_, respectively, suggesting that the radicals of •O_2_^−^ and h^+^ also contributed to the photocatalytic process. To further verify the generation of reactive species during photodegradation, ESR spectroscopy was employed during the photoexcitation process in the presence of DMPO and TEMPO spin traps. As shown in Figure 7f,g, no significant signals were detected in the absence of irradiation, confirming that no active radicals were generated under dark conditions. Upon UV irradiation, characteristic peaks corresponding to DMPO-•O_2_^−^ and DMPO-•OH were observed, displaying typical ESR signal intensity ratios of 1:1:1:1 and 1:2:2:1, respectively. In Figure 7h, the characteristic three-line ESR signal of TEMPO exhibited a significant decrease upon irradiation, confirming the generation of h^+^ species. The ESR results showed the existence of all active species (•OH, •O_2_^−^ and h^+^) during the photodegradation process of LEV.

### 2.5. Photocatalytic Mechanism

**Figure 8:**
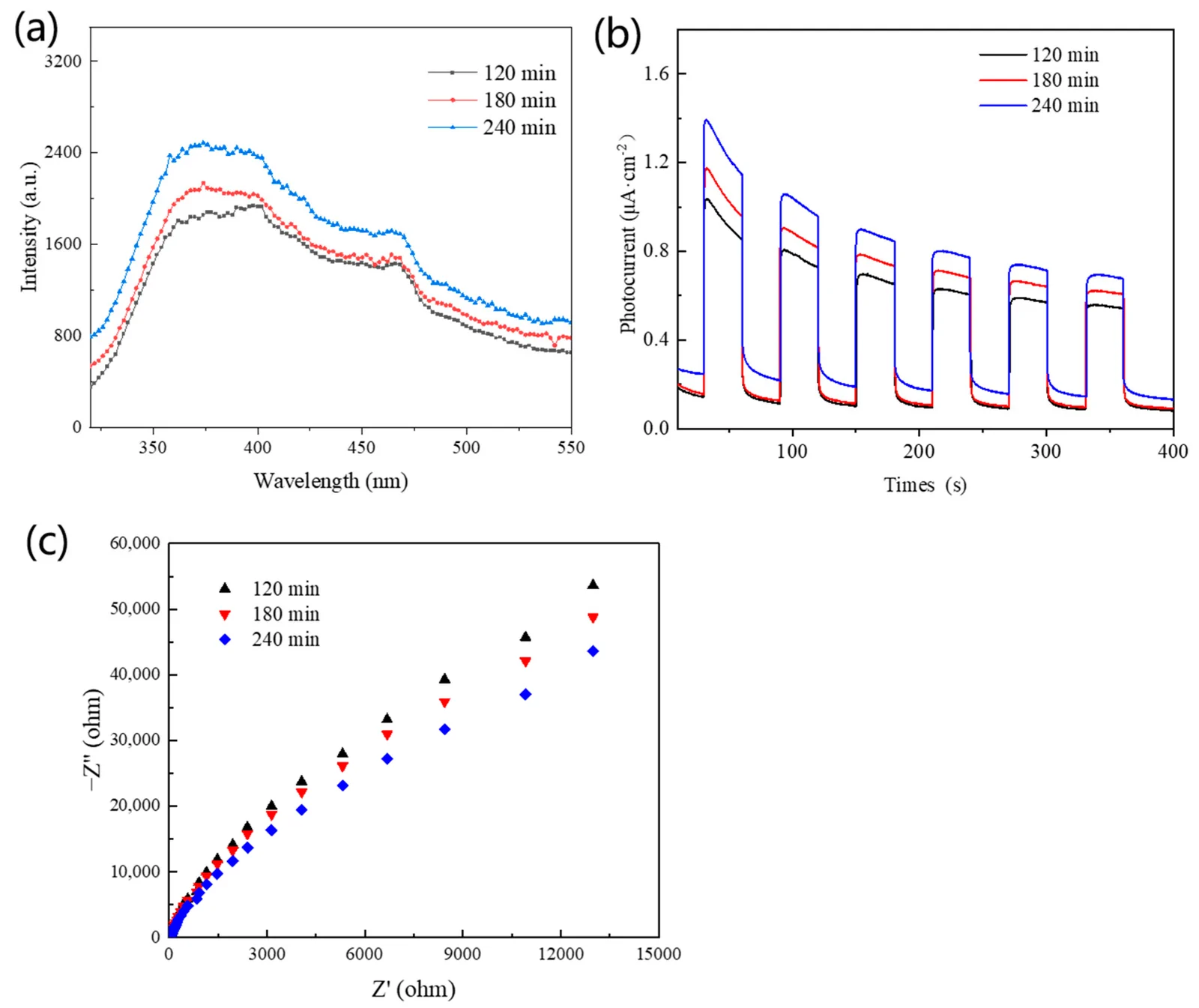
BaTi_5_O_11_ nanocrystals synthesized at 700 °C for different lengths of time: (**a**) steady-state PL spectra, (**b**) transient photocurrent responses, and (**c**) EIS Nyquist plots.

Steady-state PL spectroscopy was employed to investigate efficient charge separation and migration, which are directly correlated with the superior photocatalytic performance of the as-prepared nanomaterials. As presented in Figure 8a, the BaTi_5_O_11_ nanocrystals exhibited characteristic fluorescence peaks at 390 nm and 465 nm, corresponding to the direct and indirect recombination of photogenerated electron–hole pairs, respectively. With increasing the sintering time, the intensity of the PL peaks decreased, indicating a suppressed recombination rate of the photogenerated charge carriers [40]. BaTi_5_O_11_ nanocrystals synthesized with a sintering time of 120 min exhibited the lowest fluorescence intensity, representing the minimum recombination rate, which is consistent with their optimal photocatalytic performance, as shown in Figure 5c. To further explore the mechanism underlying the photocatalytic degradation of the BaTi_5_O_11_ nanocrystals, photoelectrochemical measurements were conducted to evaluate the separation efficiency of photogenerated electron–hole pairs. As depicted in Figure 8b, transient photocurrent (TPC) responses were recorded over six successive turning on–off irradiation cycles, with all samples displaying periodic photocurrent behavior. When the sintering time was increased, the TPC of all samples increased correspondingly. Notably, the BaTi_5_O_11_ nanocrystals synthesized with a sintering time of 240 min achieved the highest TPC value of 1.35 uA·cm^−2^ under initial irradiation, finally stabilizing at 0.69 uA·cm^−2^. Usually, a higher photocurrent response is beneficial to enhancing the photocatalytic performance, attributable to the increased carrier density and more efficient charge transfer. However, this trend contradicts the photocatalytic degradation results presented in Figure 5c. Furthermore, EIS was performed to assess the charge-transfer resistance, a critical parameter for characterizing the photoelectrochemical properties of surface-modified electrodes. The electron-transfer resistances of the samples were estimated from the diameters of the semicircles observed in the Nyquist plots, where a smaller semicircle radius corresponds to lower electron-transfer resistance at the photoelectrode surface, thus facilitating more effective separation of photogenerated electron–hole pairs. As shown in Figure 8c, the charge-transfer resistance in the Nyquist plots decreased with increasing sintering time, which again conflicts with the photocatalytic degradation results displayed in Figure 5c. The PL and photoelectrochemical spectra reveal a competing mechanism governing the effect of grain size on catalytic activity. On one hand, increasing the grain size enhances the photocurrent and reduces the charge-transfer resistance, both of which are favorable for the separation of photogenerated electron–hole pairs [41]. On the other hand, the increased grain size simultaneously exacerbates the recombination rate of photogenerated carriers. The photocatalytic degradation results in Figure 5c ultimately indicate that surface active sites and specific surface area play the dominant roles in the photocatalytic process.

**Figure 9:**
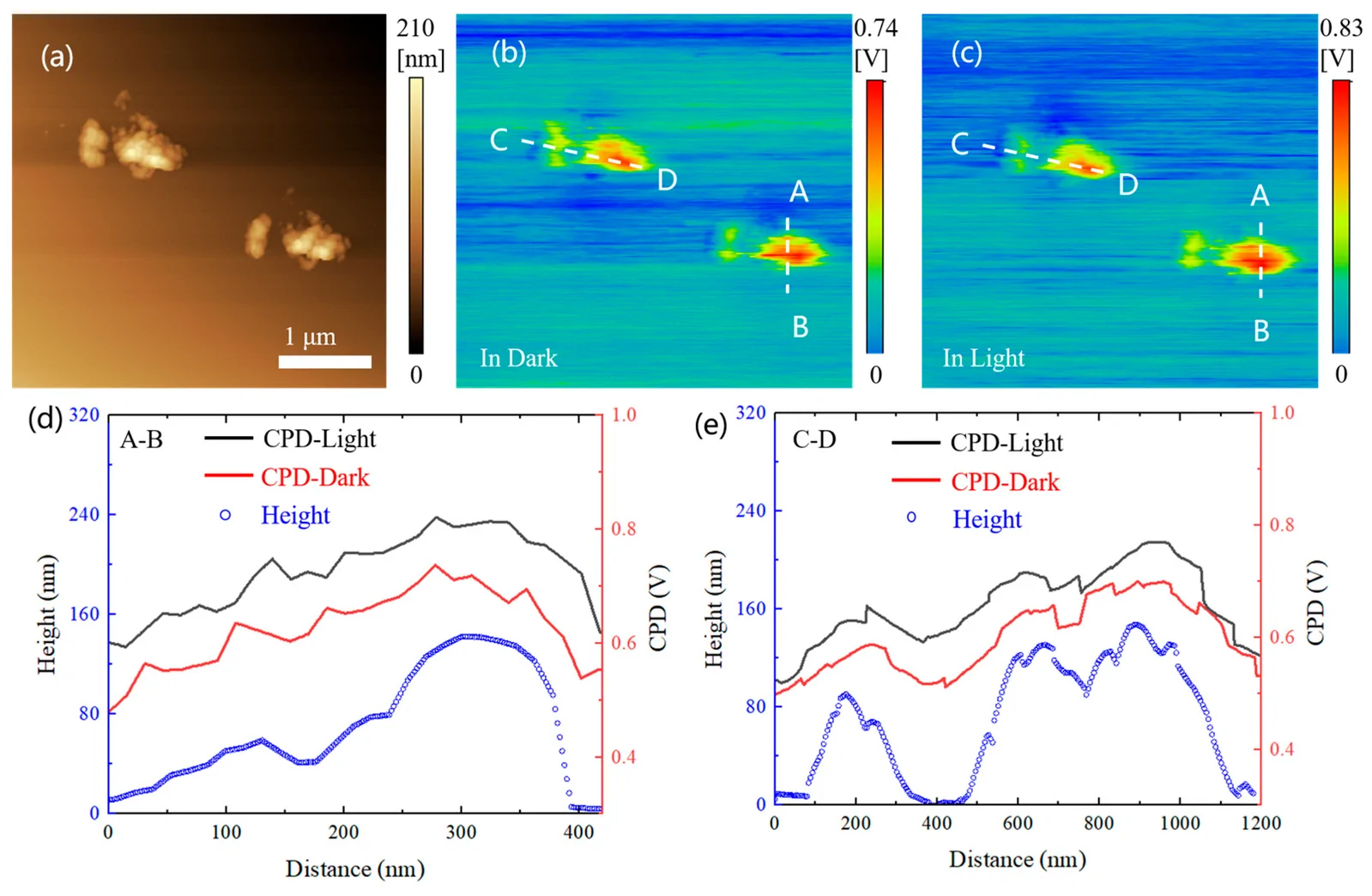
BaTi_5_O_11_ nanocrystals sintered at 700 °C for 120 min: (**a**) AFM images and (**b**) their corresponding surface potential distribution under dark state, (**c**) light irradiation, and the line profiles of surface potential and height along points A to B (**d**) and C to D (**e**).

To further elucidate the photocatalytic mechanism of BaTi_5_O_11_ nanocrystals, KPFM measurements were employed under both dark and illuminated conditions to probe the interfacial charge transfer behavior. Figure 9a shows the AFM 3D morphologies of the BaTi_5_O_11_ nanocrystals, while Figure 9b,c display the corresponding KPFM surface potential maps. Line-scan profiles acquired across the nanocrystal interface (Figure 9d,e) reveal that the maximum surface potential difference between points A and B is approximately 200 mV under dark or illumination conditions. This observation confirms the existence of a built-in electric field, which plays a critical role in the separation and transport of photogenerated electron–hole pairs [42,43]. Under light irradiation, the increase in surface potential indicates upward band bending within the space charge regions [44]. Meanwhile, the photogenerated holes migrate to the semiconductor surface, whereas electrons flow into the subsurface charge regions. The specific line-scan ΔV_CPD_ values are approximately 90 mV for the AB region and 70 mV for the CD region, suggesting that photogenerated holes can readily transfer to the crystal surface [45]. The H_2_O can be oxidized to •OH radicals by photogenerated holes for its strong oxidizing capability. According to the radical capture experiments, •OH radicals served as the primary active species responsible for LEV degradation, which is consistent with KPFM results. Furthermore, the difference in ΔV_CPD_ between these two regions may imply the exposure of different crystal facets.

## 3. Conclusions

This study systematically investigates the effect of drying temperature, thermal decomposition, and sintering conditions on the microstructure and photocatalytic performance of BaTi_5_O_11_ nanocrystals for LEV degradation, aiming to optimize efficiency without incurring additional costs. Among these parameters, sintering temperature and time critically govern the crystal morphology. At an optimized sintering temperature of 700 °C and a time of 120 min, the synthesized BaTi_5_O_11_ nanocrystals achieve a maximum LEV adsorption of 29% and a photocatalytic degradation of 93.2% within 30 min under UV irradiation, benefits that are ascribed to their high specific surface area and abundant active sites. Conversely, further increases in sintering temperature or time led to enlarged and more uniform grain sizes, which prove detrimental to photocatalytic activity. Beyond LEV, the BaTi_5_O_11_ nanocrystals also demonstrate high degradation in degrading other antibiotic pollutants, including CIP, NOF, and TC. Radical trapping experiments identify •OH as the primary reactive species contributing to LEV photodegradation. Competitive interplay between grain size and the recombination/separation dynamics of photogenerated electron–hole pairs is identified as well. Small grain size indicates a suppressed recombination rate of photogenerated charge carriers, which is attributed to that carrier being able to more easily move to the crystal surface and participate in the degradation reaction. Simultaneously, the increase in grain size enhances the photocurrent and reduces the charge transfer resistance, both of which are favorable for the separation of photogenerated electron–hole pairs. Nevertheless, the photocatalytic degradation results underscore that grain size, surface active sites and specific surface area exert the dominant influence in the photocatalytic process. This conclusion is further corroborated by KPFM measurements, which show a high cross-line ΔV_CPD_ of approximately 90 mV, indicating that photogenerated holes readily migrate to the crystal surface, thereby facilitating the generation of •OH radicals, which served as the primary active species responsible for LEV degradation.

## 4. Materials and Methods

### 4.1. Preparation of BaTi_5_O_11_ Nanocrystal

**Figure 10:**
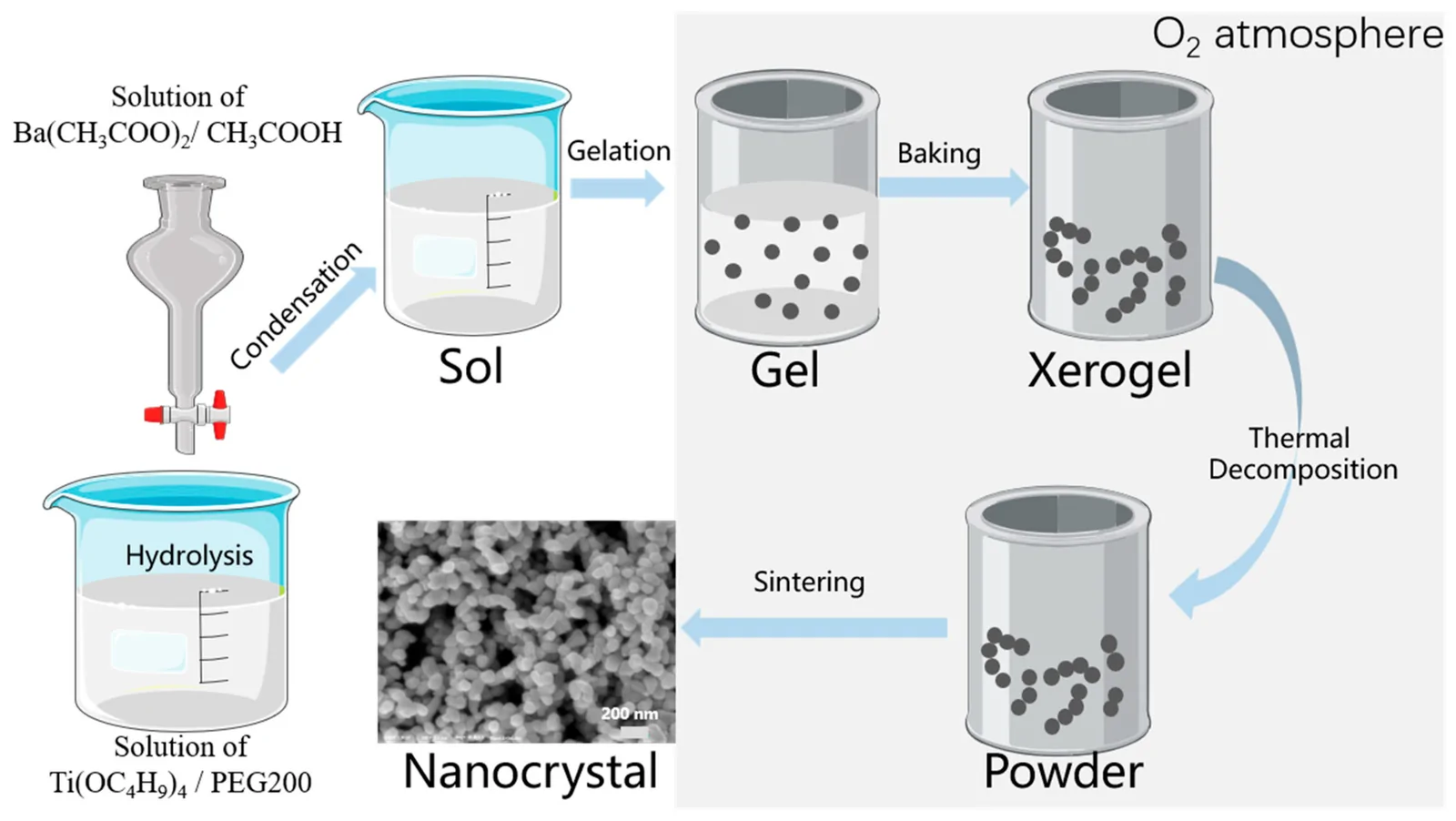
Schematic diagram of the sol–gel fabrication route for BaTi_5_O_11_ nanocrystals.

All chemicals were of analytical grade and used as received without further purification. Barium acetate (Ba(CH_3_COO)_2_, Sinopharm Chemical Reagent Co., Ltd., Shanghai, China) and tetrabutyl titanate (Ti(OC_4_H_9_)_4_, Sinopharm Chemical Reagent Co., Ltd., Shanghai, China) were employed as the sources of Ba^2+^ and Ti^4+^, respectively, with a Ba-to-Ti molar ratio of 1:5. Specifically, a predetermined amount of Ba(CH_3_COO)_2_ was dissolved in acetic acid (20 mL, Sinopharm Chemical Reagent Co., Ltd., Shanghai, China), while Ti(OC_4_H_9_)_4_ was dissolved in polyethylene glycol 200 (PEG-200, Sinopharm Chemical Reagent Co., Ltd., Shanghai, China). These two solutions were then mixed under stirring to obtain a transparent Ba–Ti precursor with a concentration of 0.1 mol/L (100 mL). BaTi_5_O_11_ nanocrystals were synthesized by the sol–gel method followed by a three-step heat treatment in a quartz tube furnace under a continuous O_2_ flow. The heating rate was set at 10 °C/min throughout all thermal steps. In the first step, the Ba–Ti precursor was dried at s set temperature (250, 260 or 270 °C) for 180 min to remove the solvents. Subsequently, the obtained product was decomposed at a higher temperature (300, 350, 400 or 450 °C) for a certain amount of time (60 min, 120 min, 180 min or 240 min) to yield amorphous Ba–Ti–O powder. Finally, the amorphous powder was sintered at temperatures ranging from 650 to 850 °C (650, 700, 750, 800, or 850 °C) for 60–240 min (60, 120, 180, or 240 min) to crystallize BaTi_5_O_11_ nanocrystals. A schematic illustration of the entire sol–gel synthesis process is provided in Figure 10.

### 4.2. Material Characterization

The crystal phase of the BaTi_5_O_11_ nanocrystals was examined using an X-ray diffractometer (XRD, D8 Advance, Bruker, MA, USA) with Cu Kα radiation (λ = 1.5406 Å) operating at 40 kV and 40 mA. The morphologies of the samples were characterized by field emission scanning electron microscopy (FESEM, Zeiss Ultra Plus, Zeiss, Jena, Germany). The active species were detected by an electron spin resonance spectrometer (ER 200-SRC, Bruker, MA, USA), with 5,5-dimethyl-1-pyrroline N-oxide (DMPO) and 2,2,6,6-Tetramethylpiperidinooxy (TEMPO) as radical scavengers for superoxide (•O^2−^), hydroxyl radicals (•OH) and holes (h^+^). Photoluminescence (PL) spectra were recorded with a fluorescence spectrophotometer (Hitachi F-7000, Tokyo, Japan). The ultraviolet–visible diffuse reflectance spectra (UV-Vis-DRS) were characterized by a spectrophotometer (UV-2550, Shimadzu, Kyoto, Japan), with barium sulfate (BaSO_4_) as the reference. Photocurrent responses and electrochemical impedance spectroscopy (EIS) measurements were performed on an electrochemical workstation (CHI 660e, CH Instruments, Shanghai, China), using a standard three-electrode system in 0.5 M Na_2_SO_4_ as the electrolyte. The distribution and migration of interfacial charges were analyzed by Kelvin probe force microscopy (KPFM, SPM-9700, Shimadzu, Japan).

### 4.3. Photocatalytic LEV Degradation

The photocatalytic activity of the prepared samples was evaluated by monitoring the degradation of LEV under UV light irradiation. A 150 W mercury lamp with an irradiation intensity of 70 mW/cm^2^ served as the UV light source. Prior to illumination, the BaTi_5_O_11_ nanocrystals (50 mg) were dispersed in an aqueous LEV solution (50 mL, 10 mg/L) and stirred continuously in the dark for 30 min to establish adsorption–desorption equilibrium between LEV and the nanocrystal surface. During the photocatalytic reaction, 2 mL aliquots were collected at 10 min intervals and subjected to centrifugation at 10,000 rpm for 5 min (repeated twice) to completely removed the BaTi_5_O_11_ photocatalyst particles. The residual LEV concentration in the supernatant was subsequently measured using a UV–vis spectrophotometer (UV1800PC, Jinghua Instruments, Shanghai, China).

## Data Availability

The original contributions presented in this study are included in the article. Further inquiries can be directed to the corresponding author.
